# Supplementary material for: Phytochemical Investigation of Three Cystoseira Species and Their Larvicidal Activity Supported with In Silico Studies
Source: Mar Drugs. 2023 Feb 10;21(2):117. doi: 10.3390/md21020117 (PMC9967941; doi:10.3390/md21020117)
Supplement: Supplementary file 1 [file marinedrugs-21-00117-s001.zip › marinedrugs-2169103-supplementary.pdf]

1. UPLC-ESI-MS analysis

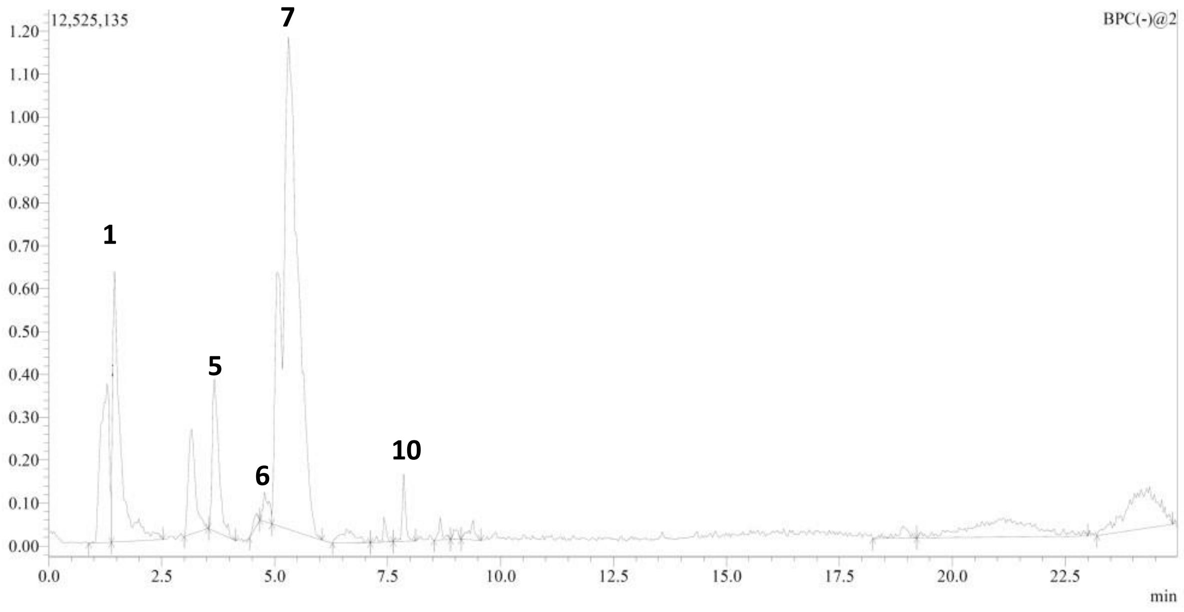

Figure S1. UPLC-ESI-MS base peak chromatogram of *Cystoseira myrica* total methanol extract in the negative ion mode.

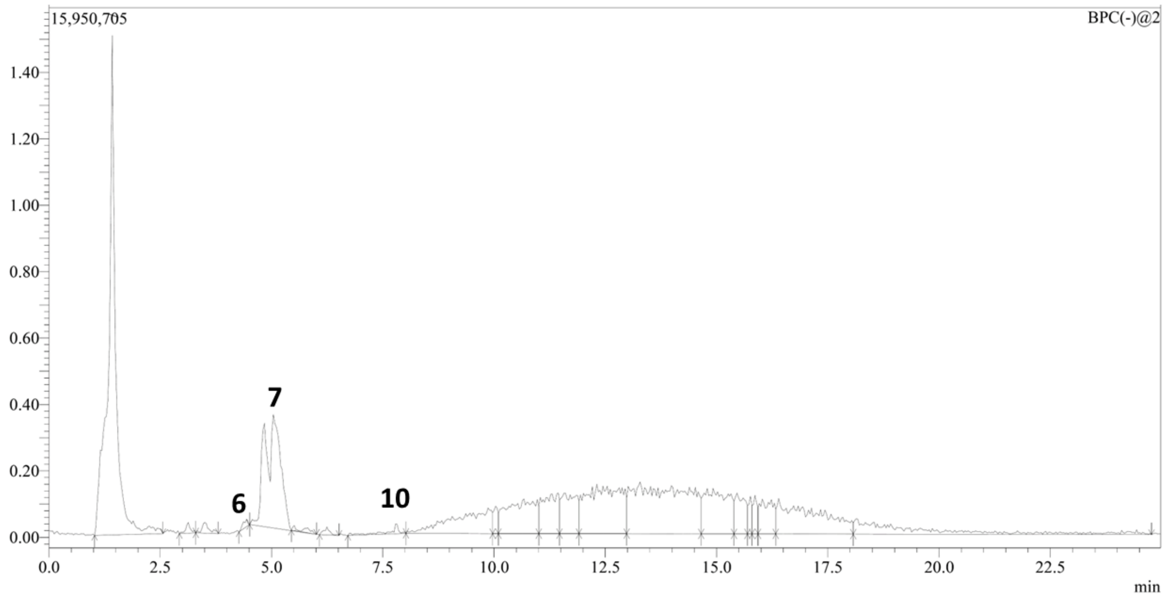

Figure S2. UPLC-ESI-MS base peak chromatogram of *Cystoseira trinodis* total methanol extract in the negative ion mode.

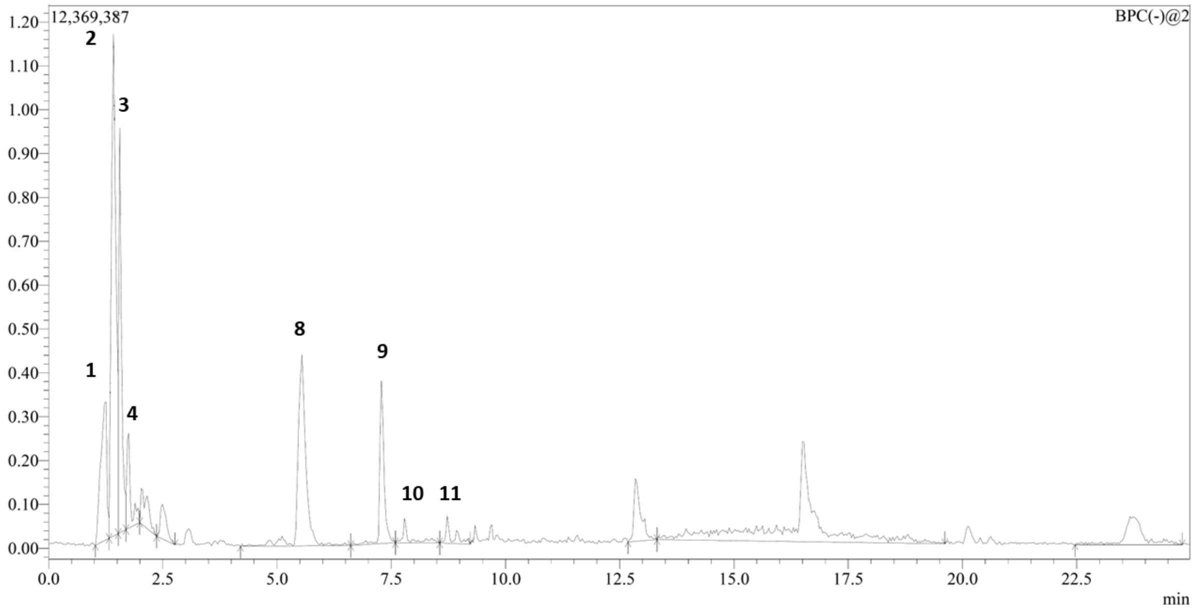

Figure S3. UPLC-ESI-MS base peak chromatogram of *Cystoseira tamariscifolia* total methanol extract in the negative ion mode.

2. Larvicidal Bioassay

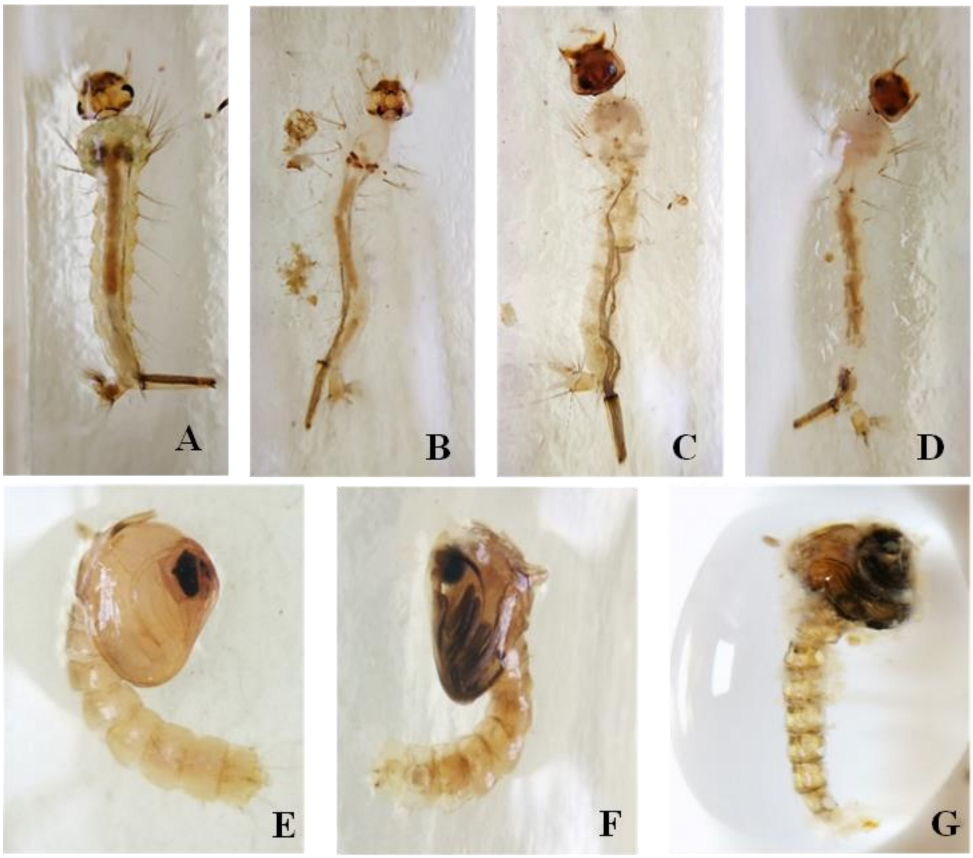

(Figure S4). *Culex pipiens* larvae with a fully developed head, thorax, abdomen, siphon, anal gill, and anal papillae (A). Abnormalities induced in larvae after treatment with *Cystoseira myrica, C. trinodis, and C. tamariscifolia* compounds showing damaged cuticle, muscle, and digestive system in the thorax and abdomen, and damaged larvae with loss of mouth brush, gill, and anal papillae (B-D). Control comma-shaped brown pupae with a well-developed cephalothorax (E). Abnormalities in pupae showing an elongated abdomen and darkened cephalothorax, highly pigmented cephalothorax and abdomen, and loss of gills (F, G).

3. In-Silico Studies

3.1. Binding mode with AChE enzyme

Compound **(4)** bound strongly to Trp321 and Asp322, respectively *via* H-arene interactions. Compound **(7)** showed good binding affinities to AChE without forming any hydrogen bonds only exposure to the receptor controls their binding effectiveness. Compound **(8)** showed weak binding modes with (BE = -4.98 Kcal/mol) to the protein pocket although it could form H-arene interaction with Tyr374. This may be attributed to its small rigid structures and minor flexible functionality. Compound **(11)** showed weak binding - binding modes with (BE = -5.59Kcal/mol) **(Figure S6)**.

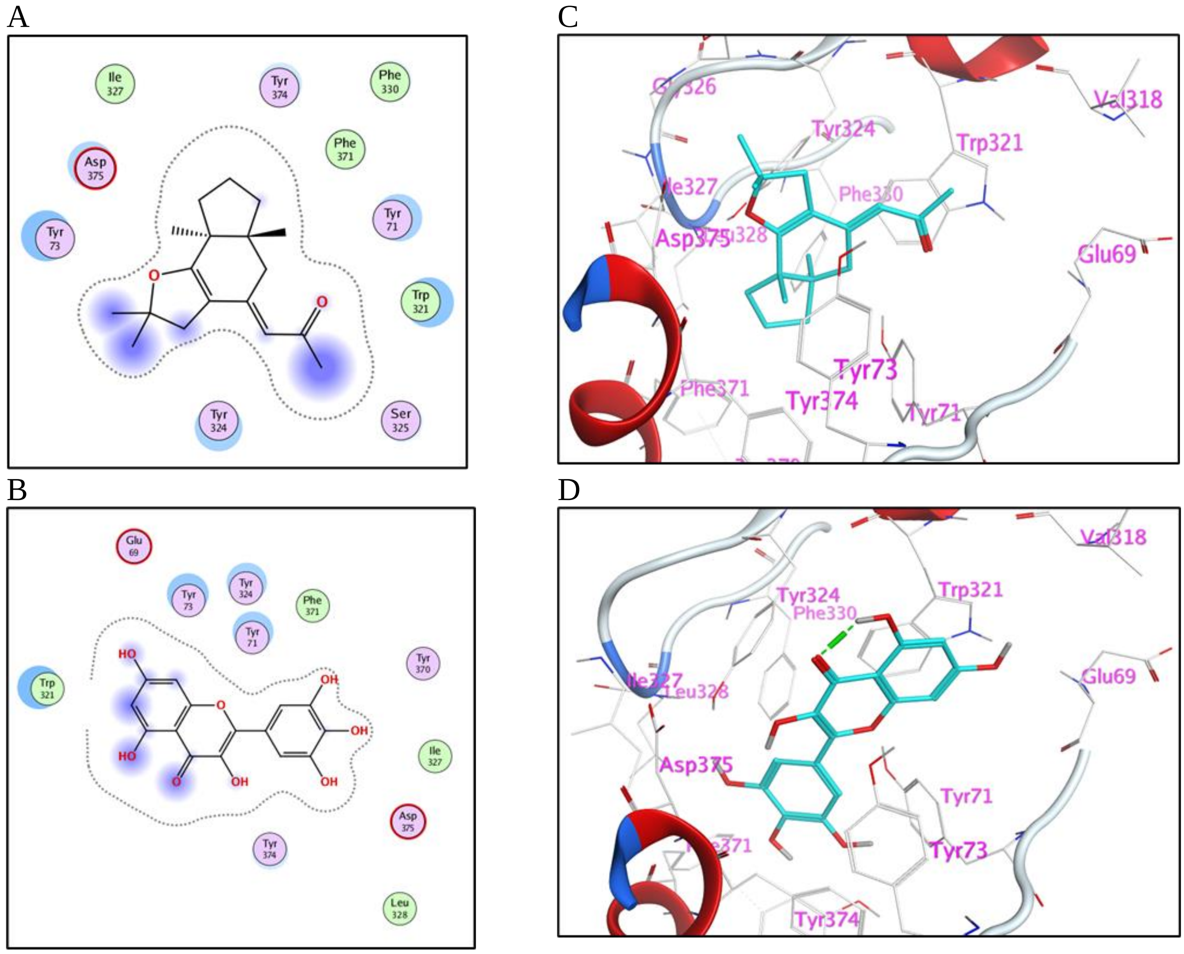

Figure S5. (**A-C**) 2D-binding interaction profile for compounds **1** and **2**, respectively, with the active pocket of AChE enzyme. (**D-F**) In-depth 3D ligand-AChE interaction mode for compounds **1** and **2** respectively.

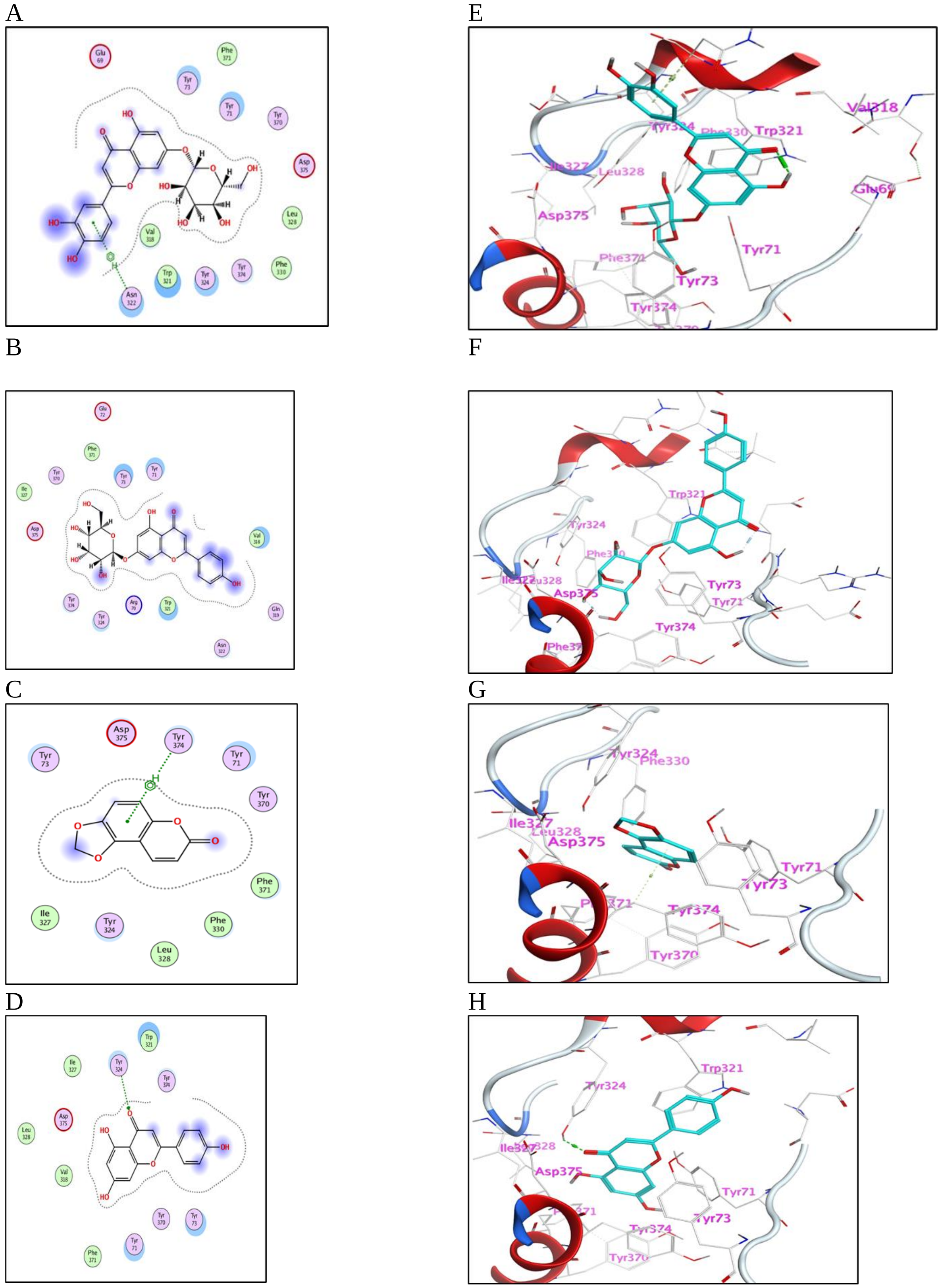

Figure S6. (**A-D**) 2D-binding interaction profile for compounds **4**,**7**,**8 and 11** respectively, with the active pocket of AChE enzyme. (**E-H**) In-depth 3D ligand-AChE interaction mode for compounds **4**,**7**,**8 and 11** respectively.

3.2. Binding mode with GST enzyme

Compounds **(1 and 8)** bind with the GST-pocket *via* forming one hydrogen bond. Among all, compound **(8)** (with Asn142) exhibited the smallest values of binding energies (-4.42 Kcal/mol) due to the same reason mentioned for binding with AChE . Besides, each of the compounds **(2, 7 and 11)** possess two hydrogen bonds with two crucial amino acid residues. Only apigenin **(11)** formed an extra H-arene interaction with Tyr211. Four hydrogen bonds control the binding of compound **(4)** with the pocket of GST, it shares the formation of H-bonds with two crucial residues, Arg145 and Tyr208 (**Figure S7 & S8)**

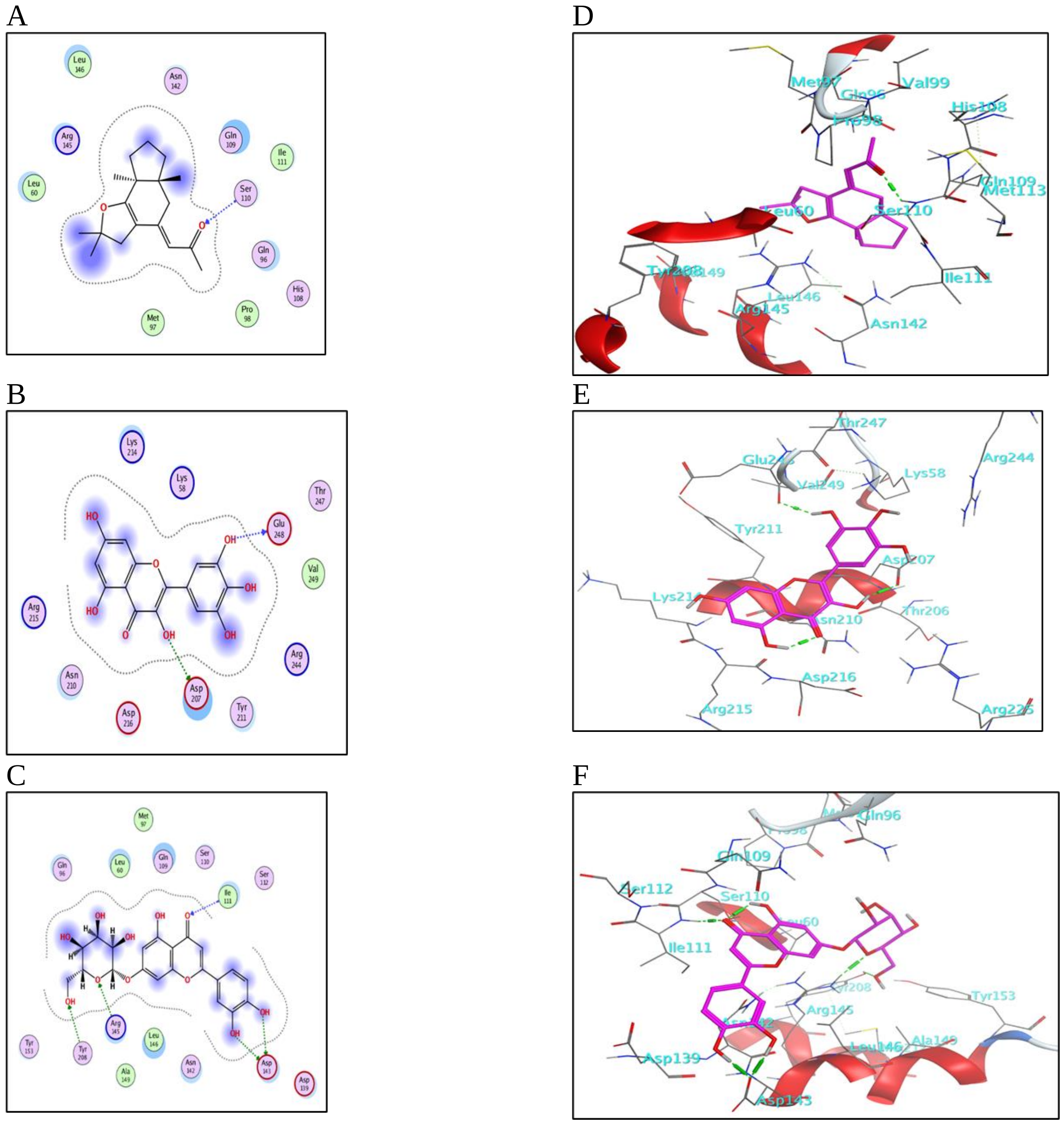

Figure S7. (**A-C**) 2D-binding interaction profile for compounds **1**, **2**, and **4**, respectively, with the active pocket of GST enzyme. (**D-F**) In-depth 3D ligand-GST interaction mode for compounds **1**, **2**, and **4**, respectively.

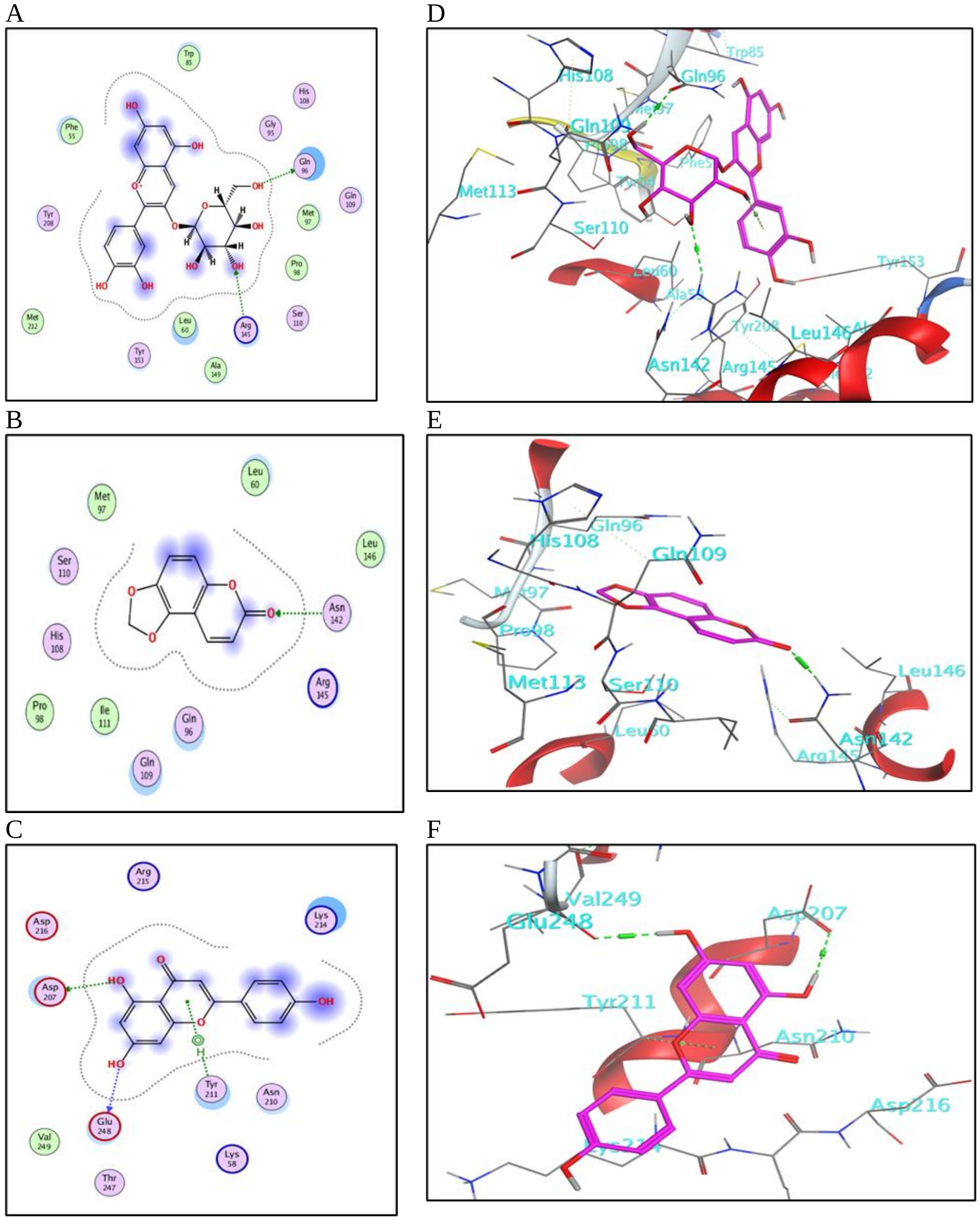

Figure S8. (**A-C**) 2D-binding interaction profile for compounds **7,8, and 11,** respectively, with the active pocket of GST enzyme. (**D-F**) In-depth 3D ligand- GST interaction mode for compounds **7**, **8**, and **11**, respectively.

A

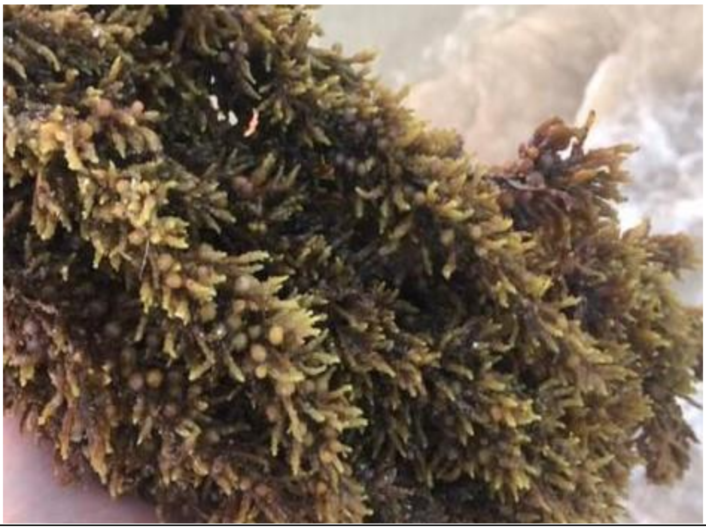

B

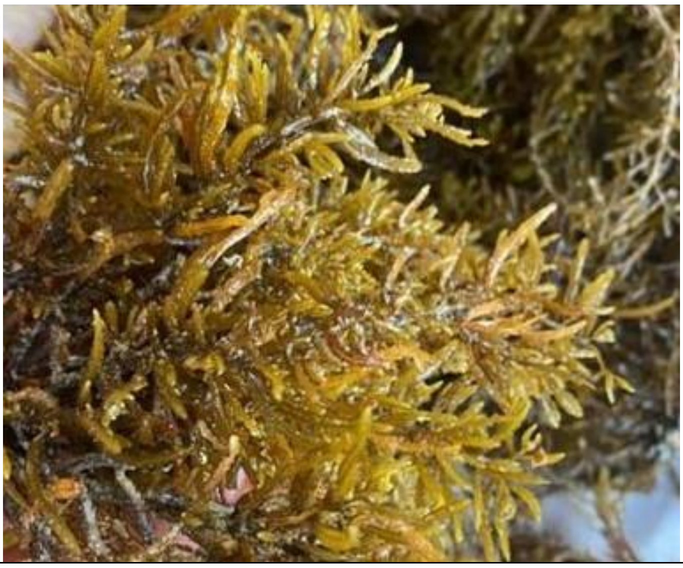

C

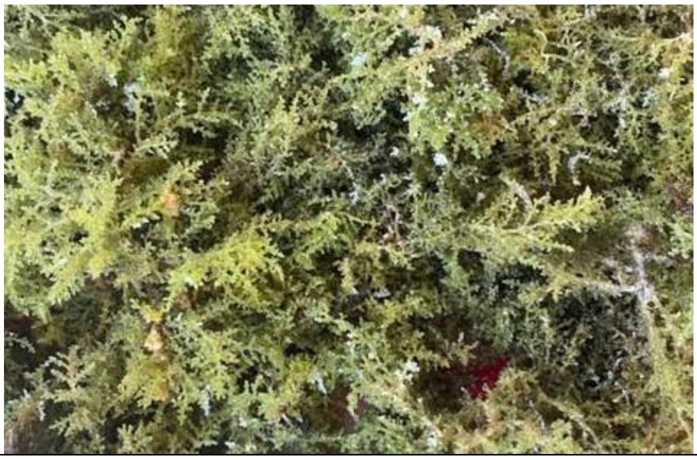

Figure S9. High-resolution images of the three samples of the work; **A.** *Cystoseira myrica*, **B.** *Cystoseira trinodis*, **C.***Cystoseira tamariscifolia*.

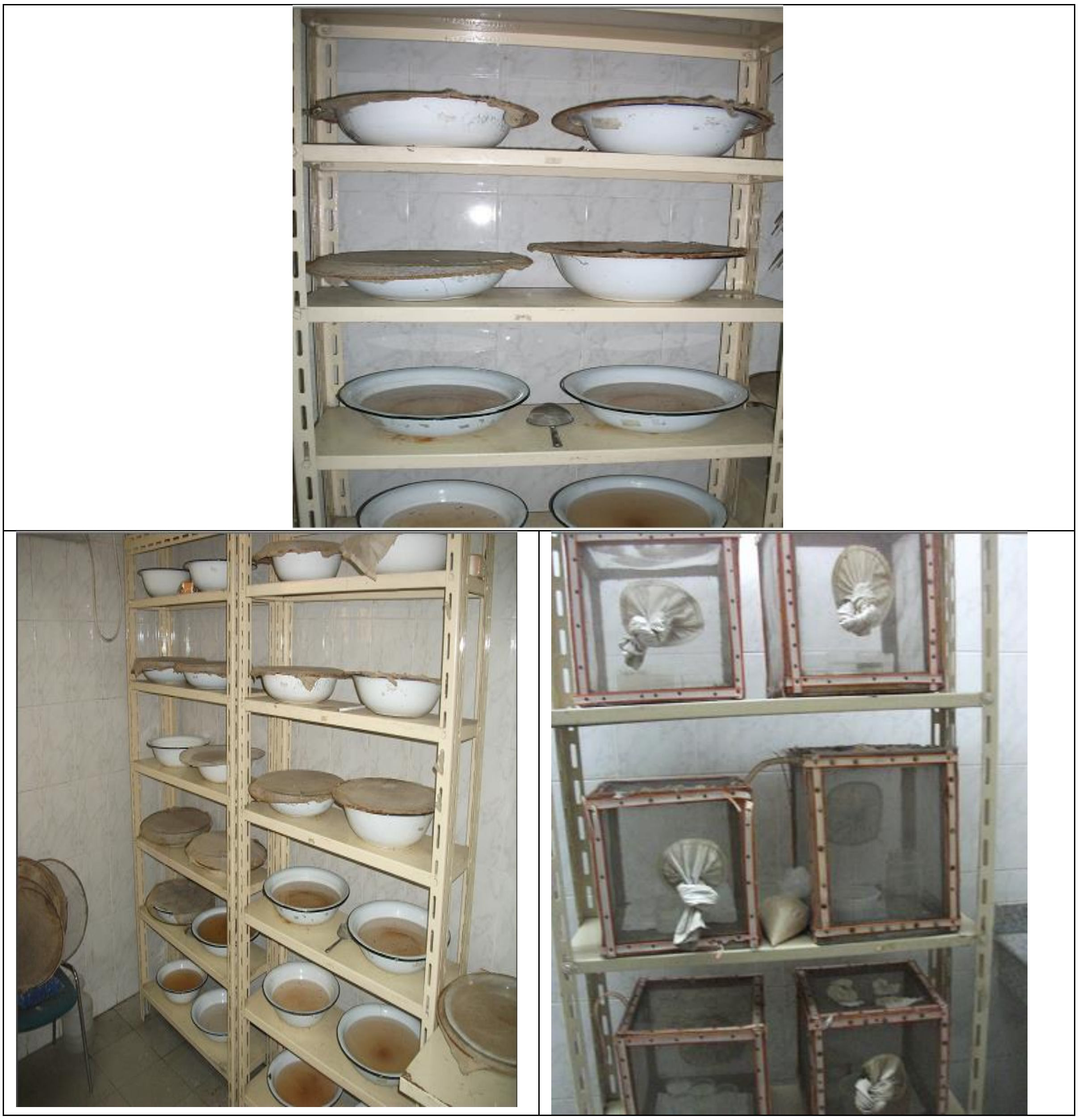

Figure S10. Laboratory Maintenance of *Cx. pipiens*.
